# Hydrothermal Processing of *Laminaria ochroleuca* for the Production of Crude Extracts Used to Formulate Polymeric Nanoparticles

**DOI:** 10.3390/md18070336

**Published:** 2020-06-27

**Authors:** Noelia Flórez-Fernández, Milena Álvarez-Viñas, Filipa Guerreiro, María Dolores Torres, Ana Grenha, Herminia Domínguez

**Affiliations:** 1 Department of Chemical Engineering, University of Vigo, Campus Ourense, As Lagoas, 32004 Ourense, Spain; noelia.florez@uvigo.es (N.F.-F.); milalvarez@uvigo.es (M.Á.-V.); matorres@uvigo.es (M.D.T.); 2 Centre for Marine Sciences, Universidade do Algarve, Gambelas Campus, 8005-139 Faro, Portugal; filiparhg@gmail.com; 3 Drug Delivery Laboratory, Faculty of Sciences and Technology, Universidade do Algarve, Gambelas Campus, 8005-139 Faro, Portugal

**Keywords:** environmentally friendly extraction technologies, bioactive compounds, polymeric nanoparticles, cytotoxicity

## Abstract

A green extraction process using only water was proposed for the simultaneous extraction of alginate and bioactive compounds from *Laminaria ochroleuca*. Operation was carried out during non-isothermal heating up to maximal temperatures over the range of 70 °C to 100 °C. Once separated, the alginate and the crude extract were characterised and the biological activities and cytotoxicity of the extracts was studied, the latter in intestinal epithelial cells. Those alginates obtained at 90 °C exhibited the highest extraction yields and viscoelastic features of the corresponding hydrogels. The obtained results show that the extracts obtained by non-isothermal extraction were suitable to formulate nanoparticles, which showed the smallest size (≈250–350 nm) when the higher content of fucoidan extract was present. Given the evidenced properties, the extracts may find an application in the formulation of nanoparticulate carriers for drug delivery.

## 1. Introduction

Traditionally, algae have been part of the diet in Asian countries and, nowadays, more countries are progressively introducing seaweeds into food applications, due to their biological properties. The commercial interest of gelling components is also increasing, and algae are great sources of such substances. *Laminaria ochroleuca* is an edible brown seaweed, also known as Kombu, with valuable nutrients such as proteins, minerals and unsaturated fatty acids [1], as well as bioactive phenolics such as phlorethols, fucophlorethols and fuhalols [2]. This seaweed further provides polysaccharides, including alginate, which is interesting for its gelling properties; laminarin, which shows immunomodulatory properties and apoptotic action in cancer cells [3]; and also fucoidans, which have increasing applications based on varied biological features [4].

**Figure 1:**
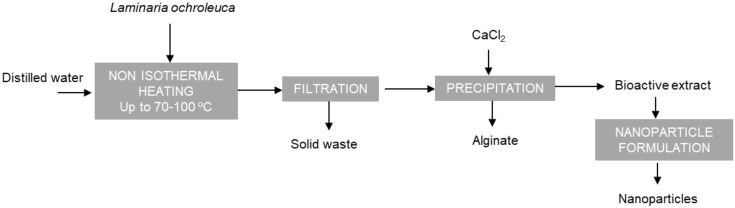
Simplified flow diagram of the aqueous process defined to obtain alginate and bioactive extract to formulate nanoparticles.

Aqueous hot extraction can be a simple, greener process, compared with those based on the use of acids or alkalis. Operation under pressurised conditions can lead to rapid extraction of both polar and non-polar compounds. In addition, the hydrolysis of biopolymers can enhance extraction yields and depolymerisation of fucoidans, which could promote their activity [5]. Moreover, alginate with similar viscoelastic features to those commercially available could be extracted by the mentioned aqueous extraction process. In a previous study, aqueous extraction under subcritical conditions and non-isothermal operation allowed the extraction and depolymerisation of fucoidan and phlorotannin fractions of *L. ochroleuca* [4]. Operating at 220 °C, a maximum extraction yield of 80% and phenolic content were obtained, but the maximal fucose and sulphate content were attained at lower temperatures. Furthermore, the apparent viscosity of the alginate recovered as a by-product was lower than for commercial solutions when the extraction temperature was increased. The process used to obtain these fractions (alginate, fucoidan) is depicted in Figure 1. Therefore, the possibility of using lower extraction temperatures during hydrothermal processing would be of interest to avoid the co-extraction of components other than fucoidans and to avoid thermal degradation.

Fucoidans have been reported to have a wide variety of biological activities [6], including antioxidant, anti-inflammatory, antifungal, antiviral, antithrombotic and antitumoural effects [7], as well as immunomodulatory properties [8]. Their composition varies according to their source (the algae of origin), geographical location, collecting season, water salinity, sun irradiation, nutrient content and other environmental factors. In this context, the brown seaweed *Laminaria ochroleuca* belongs to the order of Laminariales, to which is generally associated a structure consisting of a backbone of α(1→3)-linked L-fucopyranose residues with sulphate groups at the C-2 position [9]. The presence of sulphates in the residues has supported the use of polyelectrolyte complexation to formulate nanoparticles reported in different biomedical applications, as the ionic nature enables the formation of complexes with oppositely charged molecules. Chitosan (CS), a cationic polysaccharide extracted from the exoskeleton of crustaceans and comprised of *N*-acetylglucosamine and D-glucosamine units, has been used as the counterion [10]. Applications of fucoidan/chitosan-based nanoparticles have been reported in tissue engineering [11], wound healing [12] and drug delivery [13], benefiting from the reported absence of toxicity and the advantages related with availability, cost, biocompatibility and biodegradability reported for both polymers. The nanoparticles have revealed abilities that include the intracellular delivery of pro-oxidant agents for cancer-specific apoptosis [14] or use as adjuvants for the anthrax vaccine [15]. Fucoidan has been further reported as a component of other drug formulations, including liposomes, microparticles and semisolid formulations [16,17]. In some of the reported applications, the carriers exhibited potent antioxidant effecst [16]. The authors of this paper have reported an application of fucoidan as a matrix material of inhalable microparticles, associating two first-line antitubercular drugs, in a strategy of inhalable tuberculosis therapy. Fucoidan microparticles have shown ability to undergo internalisation by alveolar macrophages and activate the target cells [18,19].

This work is focused on the extraction of polysaccharide fractions from the edible brown seaweed *Laminaria ochroleuca,* testing different extraction temperatures. The obtained fractions were characterised and the effect on cell viability was assessed, aiming at verifying the biological properties of the extracts and their potential to prepare drug carriers with potential biomedical applications. Alginate was obtained by precipitation after the extraction process on the liquid phase for all temperatures tested and the mechanical characterisation of the respective alginate extracts was performed. In a final stage, the feasibility of fucoidan extracts to prepare polyelectrolyte complexes with CS was assessed. The obtained nanoparticles were characterised regarding physicochemical characteristics and the effect of using fucoidan extracts obtained at different temperatures on nanoparticle properties was evaluated.

## 2. Results and Discussion

### 2.1. Influence of the Final Extraction Temperature on the Yield and Extract Composition

Increased severity during the extraction process led to slightly higher solubilisation yields and corresponding solid content in the extracts (Table 1). Alginate exhibited the highest extraction yields (17.3 ± 1.2 g/100 g) at 90 °C, without statistical differences at 100 °C. However, protein yield was not enhanced with temperature in this range of operational conditions. Its content in the extract, 0.12% protein, as well as the phenolic content, 0.56%, were low compared to those attained operating at higher temperatures (200 °C), which reached 3.5% and 2%, respectively [5]. The antiradical properties were accordingly low [5], and were lower than those of sulphated polysaccharides of *L. ochroleuca* obtained by extraction at 100 °C during 135 min and selectively precipitated with 2% *O*-*N*-cetylpyridinium bromide, 4 M NaCl and 96% ethanol [8].

At the milder conditions the major monosaccharide was glucose (Table 2), which could come from laminarin, a low molecular weight polysaccharide composed of glucopyranosyl residues linked by β-1,3- and β-1,4-d-glycosidic bonds or by β-1,6-d-branched β-1,3-d-glycosidic bonds, with high solubility in water [3]. With increasing severity, the extracts were progressively enriched in the monosaccharides from the fucoidan fraction. The monosaccharide content increased due to the degradation caused in the slightly acidic media. The saccharide fraction represents around 21% at 80 °C, 38% at 90 °C, 46% at 70 °C and 64% at 100 °C.

In the range of studied temperatures, two trends can be observed. When the maximum temperature is higher than 70 °C (severity 0.04), a drop in the sulphate and monosaccharide content was observed, with the exception of rhamnose. Perhaps the increase of temperature helps the solubilisation of this hexose. However, when the severity increased to 0.25 and 0.56, the sulphate and monosaccharide contents increased, suggesting an additional solubilisation from polymeric fractions. The sulphate content was maximal at the lowest extraction temperature, possibly indicating high stability of these bonds, whereas the fucose content was maximal in the extracts obtained at the highest severity tested in this study. In a previous work by our group [5] it was reported that a maximal fucose content of 17% was attained at 180 °C, whereas the maximal sulphate (1.5%) was achieved at 160 °C, and phenolic and protein content at 220 °C (2.1%).

### 2.2. Physicochemical Characterisation of the Extracts

#### 2.2.1. Fourier-Transform Infrared Spectroscopy (FTIR)

**Figure 2:**
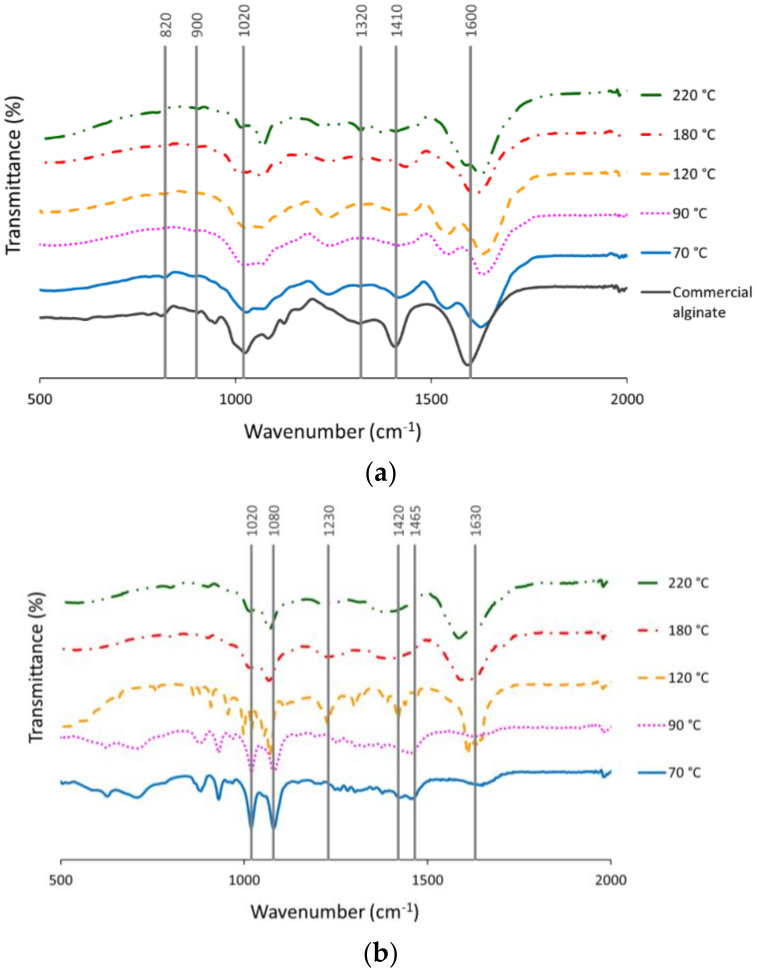
FTIR spectra of (**a**) alginates and (**b**) bioactive extracts obtained from autohydrolysis liquid extracts of *L. ochroleuca* at different temperatures.

Figure 2a shows the FTIR spectra of the alginates obtained under the different hydrothermal conditions and after the CaCl_2_ (1%) precipitation step. Bands obtained at 1600 cm^−1^ were associated with the polymeric matrix of alginate, being the carboxylate asymmetric stretching vibration O-C-O exhibited [20]. The absorption at 1400 cm^−1^ corresponds to asymmetric stretching vibrations from uronic acids. Deformations of O-C-H and C-C-H were exhibited at 1320 cm^−1^ [7]. The peak at 1020 cm^−1^, assigned to the OH bending, was widened with increasing extraction temperature, suggesting some change (increase) in the mannuronate concentration [21] and was less marked in the extracts obtained at 180–220 °C. The signal band at 900 cm^−1^ was associated with α-l-guluronic asymmetric ring vibration, whereas 820 cm^−1^ was attributed to the β-mannuronic acid residues [20].

Figure 2b shows the FTIR spectra for the bioactive extracts obtained by hydrothermal processing of *L. ochroleuca*. Different bands were observed according to the final extraction temperature. The band obtained at 1630 cm^−1^ could be correlated with aminosugars and proteins linked with the amine group [5], whereas peaks at 1420–1465 cm^−1^ were attributed to the vibrational deformation of C-OH due to symmetric stretching vibration of O-C-O (carboxylate group) [22]. Bands at 1020 and 1080 cm^−1^ could be attributed to the pyranose ring from the C-O and C-C stretching vibrations [23]. The band indicated at 1230 cm^−1^ may be related with the existence of groups of sulphate ester (S=O) [23]; lower temperatures exhibited these peaks more markedly.

#### 2.2.2. Molecular Weight Distribution

**Figure 3:**
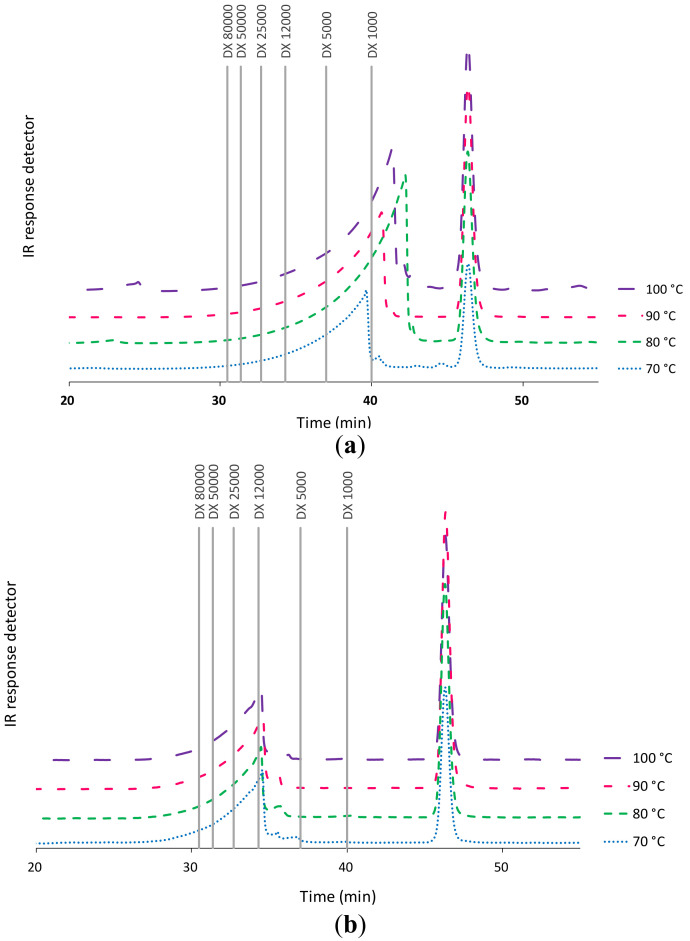
Influence of the final heating temperature during hydrothermal treatment of *L. ochroleuca* on the high performance size exclusion chromatography (HPSEC) profiles of (**a**) alginates and (**b**) bioactive extracts.

Data from high performance size exclusion chromatography (HPSEC) analyses shown in Figure 3a confirm the influence of temperature on the obtained alginate fractions. In fact, higher temperatures resulted in the generation of alginate fractions of lower molecular weight. Figure 3b shows that the severity of the hydrothermal treatment in the studied range did not significantly change the molecular weight profile of the fucoidan fraction. The chromatograms showed a broad peak corresponding to 12 kDa and a narrower one under 1 kDa. The increase in the final heating temperature during hydrothermal treatment promoted the modification of the molecular weight distributions of the corresponding alginates, suggesting the breakage of the polymer chains with the extraction temperature [5].

#### 2.2.3. Mechanical Characterisation of Alginate

**Figure 4:**
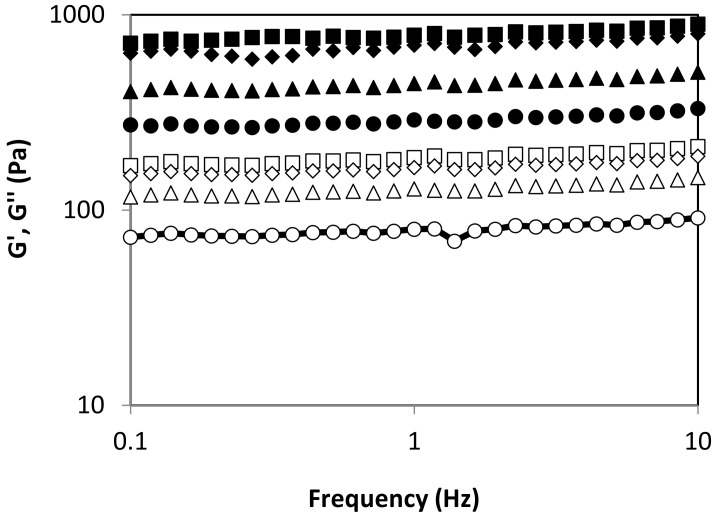
Viscous and elastic modulus for obtained alginates using different autohydrolysis temperatures: 70 (circles), 80 (triangles), 90 (squares) and 100 (diamonds) °C. Symbols: G’―closed, G’’―open.

Figure 4 shows the effect of autohydrolysis processing temperature on the viscoelastic behavior of alginates recovered from *L. ochroleuca*. All alginate-based hydrogels presented typical gel behavior, with G’ > G’’ and both moduli frequency independent. At fixed angular frequency, the elastic and viscous moduli increased with increasing autohydrolysis temperature up to 90 °C. No differences were identified between 90 °C and 100 °C. The obtained outcomes pointed out that the viscoelastic properties of tested hydrogels are highly dependent on previous thermal treatment. This suggests that the average molecular weight and molecular weight distribution is critically relevant for the mechanical features of hydrogels formulated with recovered alginates. However, the values of both moduli are consistent with those previously reported for other soft alginate matrices [24]. As an alternative to the conventional time- and chemical-consuming processes, novel alginate extraction methodologies are being developed, i.e., based on reactive extrusion, which reduces time from about an hour to a few minutes, and reduces water and reactant requirements by more than two, enhancing extraction yield and rheological properties [25]. Furthermore, alginate extracted by a pressing stage exhibited similar viscoelastic features to those that are commercially available [26]. The results of the present study provide a broad range of alginates with different viscoelastic features using only water as an extraction agent, which could be interesting to cover potential food and non-food applications.

### 2.3. Biological Characterisation and Application of the Extracts

#### 2.3.1. Effect on Cell Viability

Several authors reported that seaweed polysaccharides possess bioactive compounds linked with the identification of several potential biological activities [27,28] based on their composition and structure [6,29]. In agreement with this premise, biomedical applications have been claiming increasing importance [30,31]. Some reports have identified the antitumoral properties of fucoidan [32,33], which were also demonstrated in carrier systems composed by the polymer, such as nanoparticles and microparticles [34]. A recent work, for instance, reported nanoparticles composed by fucoidan from *Undaria pinnatifida*, showing enhanced inhibition of human pancreatic cancer cells (PANC-1 cells) [17,31]. To unveil the effect on cell viability of the crude fucoidan extracts from *L. ochroleuca* obtained herein, an in vitro cytotoxicity assay was performed. HCT-116 cells, a model of the intestinal epithelium, were used for the effect. At first, cell metabolic activity was assessed upon exposure of the cells to the extracts using 3-(4,5-dimethylthiazol-2-yl)-2,5-diphenyltetrazolium bromide (MTT), which evaluates mitochondrial metabolism [35].

**Figure 5:**
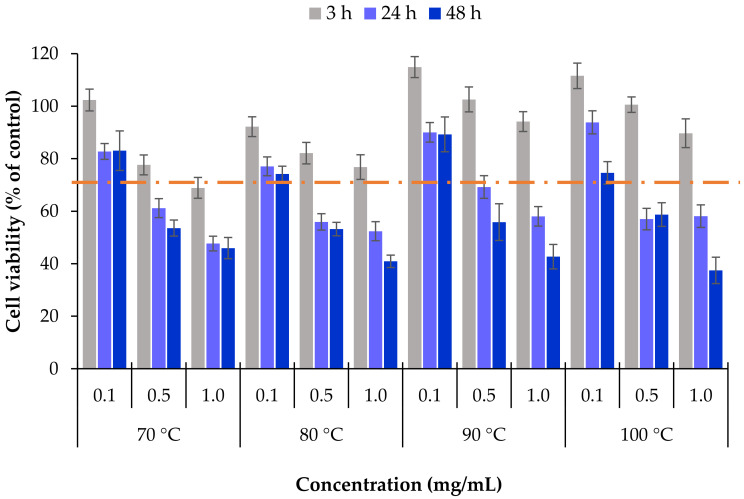
Cell viability of HCT-116 cells (intestinal epithelium) upon exposure to *L. ochroleuca* extracts obtained under different conditions of temperature extraction. Data represent mean ± SEM (*n* ≥ 3). Dashed line represents 70% cell viability.

The cells were exposed to different concentrations of the extracts and cell viability determined after 3, 24 and 48 h. Figure 5 shows the obtained results. The first relevant observation is that a short-term exposure (3 h) did not have a toxic effect for any of the tested substances, independently of the concentration. Indeed, cell viability remained in all cases close to or clearly above 70%, which is the acceptable threshold for cytotoxicity according to ISO 10993 [36]. Nevertheless, prolonging the contact of the cells with the extracts up to 24 h and 48 h elicited strong decrease of cell viability, particularly evident at the more prolonged time (*p* < 0.05), where cell viabilities around 40% were registered in some cases. Regarding the effect of the concentration, signs of toxicity occurred only at 0.5 and 1 mg/mL. Analysing individually the extracts obtained at each temperature, there is a clear effect of the concentration, with significant decrease of cell viability with the increase of the concentration (*p* < 0.05). In parallel, cell viability decreased beyond 45% upon 48 h exposure to all the extracts at the concentration of 1 mg/mL, regardless of the temperature used in the extraction process. As a whole, a general effect of time and concentration was visible on HCT-116 cells (*p* < 0.05).

The results further suggest that lower temperatures of extraction could improve the antitumoral activity of the extracts. In fact, at 24 h, the cell viability induced by the extracts obtained at 70 °C and 80 °C was frequently lower when compared with that observed upon contact with extracts obtained at 90 °C and 100 °C. This could be an effect of the extract composition, as this fucoidan was mainly comprised of fucose and sulphate groups. The maximum fucoidan:sulphate ratio was obtained at 70 °C and 80 °C (1:0.61 and 1:0.50 respectively), compared with 1:0.15 and 1:0.12 for 90 °C and 100 °C, respectively.

However, this effect vanished after 48 h, when cell viability was quite similar in all samples, with the lowest nominal value of 37% cell viability being obtained for the extract from 100 °C. Interestingly, this sample corresponds to that possessing the maximum percentage of fucose content (Table 2). The effect could thus be attributed to a cytotoxic action of fucoidan, as reported in other works [33,37]. A previous study reported on the inhibitory capacity of extracts of *L. ochroleuca* in A549 cells, from lung adenocarcinoma, and in the same HCT-116 cells, but that work tested extracts obtained at higher temperatures. Extracts obtained from 120 °C to 220 °C required a concentration of 25 µg/mL to reduce cell viability to 50% [5]. However, we did not test such concentrations in the present work, as the extracts have lower fucoidan contents when produced at lower temperatures. The minimum extract concentration tested herein was 0.1 mg/mL, which did not result in cell viability reduction. The difference in the observed results is certainly an effect of the composition of the extracts, because at higher temperatures such as 120–220 °C the polysaccharides are highly degraded and, consequently, the oligosaccharide content is very low. Nevertheless, the extract obtained at 200 °C and 220 °C showed a high phenolic content and Trolox equivalent antioxidant capacity. On the other hand, sulphated polysaccharides (0.6% S (sulfur) content and 48.6 C/N ratio) of *L. ochroleuca* were also reported to reduce the cell viability of HTC-116 cells to 50% at a concentration of 44 µg/mL, but required more than 1 mg/mL for other human cancer cells [8].

**Figure 6:**
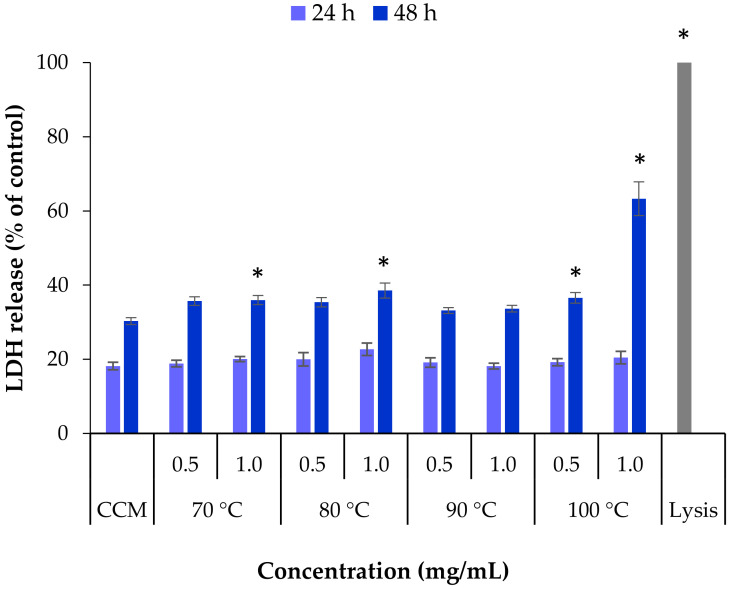
Lactate dehydrogenase (LDH) released from HCT-116 cells after 24 h and 48 h exposure to *L. ochroleuca* extracts obtained under different extraction temperatures. Data represent mean ± SEM (*n* ≥ 3). * *p* < 0.05 compared with the negative control (cell culture medium, CCM).

Complementarily to the evaluation of mitochondrial activity, the determination of the effect of exposure to extracts on the integrity of the cell membrane was further investigated as another indicator of toxicity. To do so, the release of the cytoplasmic enzyme lactate dehydrogenase (LDH) was quantified [38,39]. The extracts obtained from *L. ochroleuca* at different temperatures were tested at the two highest concentrations used in the MTT assay, 0.5 and 1.0 mg/mL, and exposure of HCT-116 cells for 24 h and 48 h (Figure 6).

The basal release of LDH from cells incubated with cell culture medium only was used as negative control. The positive control (Triton X-100, lysis buffer) was assumed as having 100% release of LDH, which was, as expected, higher than that obtained for all the samples (*p* < 0.05). After 24 h none of the extract samples induced a significant release of LDH compared with the negative control CCM. Extending the exposure time to 48 h resulted in a significant increase in the released LDH for some samples (*p* < 0.05), although generally it was not very intense. The effect induced by the extract obtained at 100 °C, however, deserves a specific mention, as the concentration of 1 mg/mL resulted in 63% LDH release, contrasting with the 30% of the control (*p* < 0.05). This is coincident with the 37% cell viability detected in the MTT assay, indicating a clear condition of toxicity that is perhaps a result of the high content of fucose.

There were some differences between the two assays used to evaluate the cytotoxicity of the samples extracted from *L. ochroleuca* at different temperatures. The cell viability decrease observed in the MTT assay after 24 h in some cases was not expressed in the LDH assay, where this exposure time did not reveal alterations compared with the negative control. However, when the exposure was extended to 48 h, the released LDH increased in all samples, in some cases to a significantly different level. The set of assays thus suggests that extracts obtained by subcritical water extraction of the edible brown seaweed *L. ochroleuca* could be used to obtain an antitumoural effect, but the studies should be extended to draw conclusions on the adequate concentration to have such an effect. A minimum concentration of 1 mg/mL should apparently be used, but perhaps it needs to be increased. Additionally, from the tested extraction temperatures, using 100 °C seems to be the most adequate to ensure high fucose content, which links with the observed cytotoxic effect.

#### 2.3.2. Production and Characterisation of Nanoparticles Formulated with Extracts from *L. ochroleuca*

In line with the observations of the cytotoxic properties provided in the previous section, it was considered adequate to determine the ability of the crude extracts of fucoidan (FUCe) obtained herein to formulate nanoparticles, which could find an application in anticancer drug delivery in future studies. The various FUCes were thus used to prepare nanoparticles, which were formed instantaneously by complexation with CS, in the sequence of the electrostatic interactions occurring between chitosan amino groups (positively charged) and sulphate groups of the FUCe (negatively charged). Two different protocols were tested to formulate the nanoparticles, varying the order of addition of the two polymeric solutions. In one case, CS was poured over a solution of FUCe, and the obtained nanoparticles were named FUCe/CS nanoparticles. The opposite involved pouring FUCe over a solution of CS, and the resulting carriers received the designation of CS/FUCe nanoparticles. The polymeric solution receiving the other one was always at the concentration of 1 mg/mL, whereas the concentration of the pouring solution was adjusted to reach the desired mass ratio. For each protocol, nanoparticles with polymeric mass ratios corresponding to 4/1, 1/1 and 1/4 were produced, and the effect of the order of addition tested regarding nanoparticle properties. As a result of the set mass ratios, nanoparticles FUCe/CS = 4/1 are similar to CS/FUCe = 1/4, although the concentrations of the involved polymeric solutions used are different.

**Figure 7:**
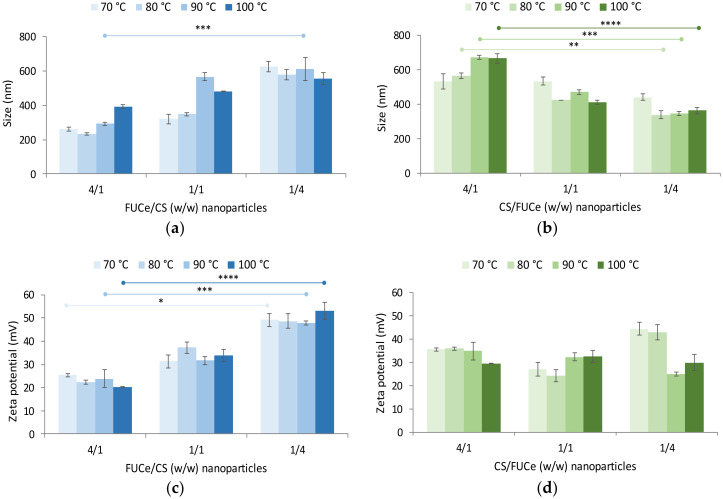
Effect of crude extracts of fucoidan/chitosan FUCe/CS (blue) and CS/FUCe (green) mass ratio on (**a**,**b**) size and (**c**,**d**) zeta potential of nanoparticles formulated using extracts obtained by hydrothermal extraction at different maximal temperatures. Data represent mean ± SD (*n* ≥ 3). Significant differences were represented by * for 70 °C, ** for 80 °C, *** for 90 °C, **** for 100 °C (*p* < 0.05), compared between ratios.

Figure 7a,b show the size of the developed nanoparticles. In FUCe/CS nanoparticles, sizes varied within 233 and 627 nm (PdI from 0.29 to 0.58, Table S1), whereas CS/FUCe nanoparticles ranged within 338 and 673 nm (PdI from 0.28 to 0.69, Table S1). The size distribution of nanoparticles is represented in Figures S1 and S2 for FUCe/CS and CS/FUCe formulations, respectively. As a general trend, smaller nanoparticles were obtained when a lower amount of CS was present, with FUCe prevailing as per the mass ratios (FUCe/CS = 4/1 and CS/FUCe = 1/4). This was accompanied by lower polydispersity index also observed for nanoparticles with lower CS amounts (Table S1). This is possibly a consequence of the molecular weights of the involved polymers. In Section 2.2.2. fucoidan was determined to have a molecular weight around 12 kDa, whereas the used chitosan has been reported to have a much higher value, around 169 kDa [40]. Naturally, complexation involving higher amounts of chitosan results in larger nanoparticles due to the presence of higher amounts of the polymer with larger polymeric chains. Moreover, comparing the corresponding ratios, FUCe/CS nanoparticles exhibited slightly lower size in most cases. These FUCe/CS nanoparticles showed an increase in size when chitosan amount increased, which was visible inclusive in the ratio 1/1 at the two higher temperatures of extraction. This could be explained by the fact that at these temperatures there are more fucose chains available to interact with chitosan, thus leading to larger particles. In CS/FUCe nanoparticles (Figure 7b), some size differences were found among the tested mass ratios and a similar pattern was observed, with size decreasing as fucoidan was incorporated and became prevalent. Disregarding the protocols of nanoparticle preparation, the temperature used for the extraction was generally found not to influence the size of nanoparticles.

Nanoparticles of lower sizes are typically preferred, as they may provide more intimate contact with cellular structures, which may result in improved therapeutic response, including cytotoxic effect. In this work, the formulation of nanoparticles of lower sizes (around 200–250 nm) was favoured when the protocol involved pouring chitosan over the fucoidan extract while simultaneously having a predominant amount of fucoidan (FUCe/CS = 4/1). Other works presented similar results for the size studied [14]. As a whole, the results show a significant effect from the order of addition of the solutions, the prevalence of one of the polymers and the mass ratios to a certain extent, whereas no effect is observed as a consequence of different extraction temperatures.

Figure 7c,d show the effects on zeta potential, presenting a variation within +20 and +53 mV for FUCe/CS nanoparticles, and between +24 and +45 mV for CS/FUCe nanoparticles. The first observation of relevance is the fact that all nanoparticles exhibited a positive charge, even in formulations where there was strong predominance of the crude extract, potentially presenting negative charge due to the presence of sulphate groups. This is a clear indicator of the higher charge density per mass unit of chitosan. Nevertheless, significantly lower zeta potential was observed when higher amount of fucoidan was present on FUCe/CS nanoparticles (4/1, w/w) (*p* < 0.05), which is a result of the neutralisation of chitosan amino groups by the sulphate groups of the extract. However, it was interesting to notice that such a clear effect was not observed in CS/FUCe nanoparticles for any of the extracts. In this case, CS was used at the fixed concentration of 1 mg/mL and fucoidan extract solutions at the necessary concentrations to reach the defined ratios were poured over CS. The absence of effect on zeta potential further reinforces the higher density of CS charge density and one possible justification could be the fact that even at the 1/4 ratio, sulphate groups of fucoidan are not enough to complex the chitosan amino group sufficiently to induce an alteration in the surface charge of nanoparticles. Moreover, as there is a great difference in the size of the polymeric chains, the smaller fucose chains may perhaps find difficulties in making their way through the large chain of CS to access the surface of nanoparticles and thus interfere with surface charge. There is an additional hypothetical explanation, relying on the previous addition of a great amount of Ca^2+^ ions for alginate extraction, which could affect the ionic strength of the nanoparticle dispersion and cause perturbations in the electrical double layer.

The obtained results thus confirm the ability of the fucoidan crude extracts obtained herein to prepare nanoparticles by polyelectrolyte complexation. It was also demonstrated that several aspects of the formulations can be adjusted to tailor the physicochemical characteristics of nanoparticles.

## 3. Materials and Methods

### 3.1. Raw Materials

*Laminaria ochroleuca* was purchased from Algamar (Pontevedra, Galicia, Spain). The brown seaweed was collected in June 2015, and was subsequently cleaned and oven dried at 42 °C. At the laboratory, the seaweed was ground and stored in hermetic plastic bags at room temperature in darkness until use.

### 3.2. Hydrothermal Extraction

The dried and ground seaweed was suspended in distilled water at a solid:liquid ratio (SLR) of 1:30 (w/w) and was then heated up to four different final temperatures (70 °C, 80 °C, 90 °C and 100 °C) in a stainless-steal pressurised reactor (Parr Instruments, series 4842, Moline, IL, USA). When the reactor achieved the set-point temperature, a quick cooling down to room temperature was carried out. Two phases were obtained after a filtration step, liquid and solid phase. Both phases were analysed and characterised.

The severity factor of the extraction process was evaluated using *L. ochroleuca* as raw material [41]. Data acquisition of two variables (temperature and reaction time) was necessary to calculate this parameter following Equation (1):

(1) $$Ro=\int_{0}^{t}\exp\frac{T\left(t\right)-Tr}{w}\;dt$$

where *R*_0_: severity factor; *T*: temperature (°C); *T*r: reference temperature (°C); *t*: reaction time and *W*: activation energy of reaction (constant = 14.75).

### 3.3. Alginate Extraction

The alginate fraction solubilised in the liquid phase obtained after hydrothermal extraction was precipitated by adding 1% of calcium chloride (w/v) (Scharlau, Barcelona, Spain) and stirring gently at least 12 h at 300 rpm (MMH90E, OVAN, Barcelona, Spain). A centrifugation step, necessary to separate the alginate fraction from the alginate-free liquid fraction, was carried out for 20 min at 4500 rpm (Rotixa 50RS, Hettich Zentrifugen, Tuttlingen, Germany).

### 3.4. Analytical Methodology

#### 3.4.1. Composition of the Extracts

##### Carbohydrate Content

The samples obtained at different temperatures during hydrothermal treatments were filtered through 0.45 μm membranes and dialysed with a membrane tubing (Spectra/Por Float-A-Lyzer G2, MWCO 0.5 kDa, SpectrumLabs, Rancho Domínguez, CA, USA). Glucose (Glu), xylose (Xyl) + galactose (Gal)+ mannose (Man), rhamnose (Rha), fucose (Fuc) and acetyl groups were analysed by HPLC. The equipment used was a Hewlett-Packard chromatograph (1100 series) with an IR detector and a 300 × 7.8 mm Aminex HPX-87H column (BioRad, Hercules, CA, USA) operating at 60 °C using 0.003 M H_2_SO_4_ at 0.6 mL/min as mobile phase. The oligosaccharide content was determined after quantitative posthydrolysis with sulfuric acid (4%) at 121 °C for 20 min. The experiments were made at least in triplicate.

##### Phenolic Content

The total phenolic content was expressed as phloroglucinol content after spectrophotometric determination following the methodology reported previously [42]. The protocol requires the addition of Folin–Ciocalteu reagent (1 N) and 20% Na_2_CO_3_ to the samples, using distilled water as a blank. After incubation at room temperature for 45 min in darkness, absorbance was read at 730 nm in a spectrophotometer (Evolution 201 UV-Visible, Thermo Scientific, Waltham, MA, USA). The calibration curve was made using phloroglucinol (Sigma-Aldrich, St. Louise, MO, USA) as standard. The measures were made at least in triplicate.

##### Antioxidant Activity

Antiradical properties of *L. ochroleuca* liquid samples, obtained by hydrothermal treatment, were determined at least in triplicate as the ABTS radical cation (ABTS^·+^) (2,2-azinobis(3-ethyl-benzothiazoline-6-sulfonate)) scavenging capacity [43]. The results were expressed as TEAC value (Trolox equivalent antioxidant capacity). Briefly, samples (10 µL) were incubated for 6 min at 30 °C with diluted ABTS^·+^ solution (1.0 mL). The blank was made with water (10 µL) and the standard curve was prepared with Trolox (6-hydroxy-2,5,7,8-tetramethylchroman-2-carboxylic acid). The absorbance was read at 734 nm using an Evolution 201 UV-Visible (Thermo Scientific, USA) spectrophotometer.

##### Soluble Sulphate Content

The gelatine-barium chloride method [44] was proposed to measure the sulphate content after hydrolysis of liquid samples with trichloroacetic acid (TCA) (4%) (Sigma-Aldrich, Barcelona, Spain). In order to prepare the gelatin-BaCl_2_ reagent, gelatine powder (Scharlau, Spain) was dissolved in hot water (70–80 °C) to reach 0.5% concentration, the solution was kept at 4 °C for at least 6 h, and then BaCl_2_ was added (0.5%). A cloudy reagent was obtained, and in 2–3 h the solution was ready. Briefly, liquid samples or distilled water for blank (0.2 mL) were added into test tubes and TCA solution at 4% (3.8 mL) and gelatine-BaCl_2_ reagent (1 mL) were added and mixed with a vortex after an incubation for 15 min at room temperature. Then, the absorbance was read at 500 nm.

##### Soluble Protein Content

Soluble protein content was spectrophotometrically determined following the protocol developed by Bradford [45]. Liquid samples from hydrothermal extraction of raw material were mixed with Bradford reagent (Panreac, Barcelona, Spain) and incubated for 5 min at room temperature. The absorbance was measured at 595 nm against a standard curve prepared using bovine serum albumin (Sigma, St. Louise, MO, USA).

#### 3.4.2. Chemical Characterisation of Extracts

##### Fourier-Transform Infrared Spectroscopy

The liquid phases obtained by hydrothermal extraction were lyophilised, blended with potassium bromide (KBr) and analysed. The equipment used was a Nicolet 6700 (Thermo Fisher, USA) with IR and DTGS KBr detectors. The software used was OMNIC. Samples were studied in a range from 400 to 4000 nm, with the spectral resolution: 4 cm^−1^ and 32 scans/min. The FTIR spectra were recorded at least in duplicate for all samples.

##### Molecular Weight Distribution

The molar mass distribution of the liquid samples obtained by autohydrolysis was studied at least in duplicate. Analyses were performed in an Agilent 1100 equipment, with two columns in series, 300 × 7.8 mm TSKGel G2500PW_XL_ and G3000PW_XL_ from Tosoh Bioscience (Griesheim, Germany) and a PW_X_-guard column (40 × 6 mm). The operation conditions were: 70 °C using Milli-Q water at 0.4 mL/min as mobile phase. To evaluate the molar mass distribution, dextrans (1000–80,000 g/mol) (Fluka, St. Gallen, Switzerland were used as standards.

#### 3.4.3. Biological Characterisation and Application

##### Effect of Extracts on Cell Viability

###### Cell Culture

HCT-116 cells (human colorectal carcinoma) were obtained from the ATCC (Rockville, MD, USA) and used between passages 6–9. Cell cultures were grown using 75 cm^2^ flasks in a humidified 5% CO_2_/95% atmospheric air incubator at 37 °C. Cells were grown in Dulbecco’s modified Eagle’s medium (DMEM) (Sigma-Aldrich, Taufkirchen, Germany) supplemented with fetal bovine serum (FBS; 10%, v/v) (Gibco, Life Technologies, Carlsbad, CA, USA) and penicillin/streptomycin (1%, v/v) (Sigma-Aldrich, Taufkirchen, Germany). Cell culture medium (CCM) was exchanged each 2–3 days and cells were subcultured weekly.

##### Evaluation of Metabolic Activity

The MTT assay was performed to evaluate the metabolic activity of HCT-116 cells after exposure to the crude extracts of fucoidan from *L. ochroleuca*. These were tested at concentrations of 0.1, 0.5, 1.0 mg/mL for exposure times of 3, 24 and 48 h, and active metabolism was assumed as cell viability. To perform the assay, HCT-116 cells were seeded in 96-well plates at a density of 1 × 10^4^ cells/well and incubated at 37 °C in 5% CO_2_/95% atmospheric air. After 24 h, the culture medium was replaced by 100 µL of the extracts solubilised in CCM without FBS. CCM was used as a positive control of cell viability and sodium dodecyl sulphate (SDS, 2%, w/v) was used as a negative control. After the predetermined exposure times, the samples were removed from the plates and 30 µL of MTT (5 mg/mL in PBS pH 7.4, Sigma-Aldrich, Taufkirchen, Germany) were added to each well. After 2 h incubation, the formazan crystals resulting from MTT metabolisation were solubilised with 50 µL of dimethyl sulfoxide (DMSO). The plates were read by spectrophotometry (Infinite M200; Tecan, Grödig, Austria) at 540 nm and corrected for background at 640 nm. Cell viability was calculated by the following equation:

(2) $$Cell\;viability=\frac{A-D}{CM-D}\;\times \;100$$

where *A* is the absorbance obtained upon exposure to each extract, *D* is the absorbance measured for DMSO and *CM* is the absorbance read for the cells incubated in CCM. The reading obtained for *CM* was assumed as 100% cell viability. The assay was carried out more than 3 times with more than 3 replicates for each concentration.

##### Evaluation of Cell Membrane Integrity

The LDH released by the cells upon exposure to the extracts was determined to study the cell membrane integrity. This assay was performed simultaneously with the MTT, using the supernatant of HCT-116 cells upon exposure to the samples. After 24 h and 48 h of exposure, the cell supernatants exposed to the higher concentrations (0.5 and 1 mg/mL) of the extracts were collected and centrifuged at 16,000× *g* for 5 min. A similar procedure was done for the controls. CCM was used as a negative control of LDH release, and triton X-100 (1:10 dilution; Amresco, Solon, OH, USA) was used as a positive control. The released LDH was determined using a commercial kit (Takara, Japan), following the supplier instructions. LDH release was quantified by spectrophotometry at 490 nm and background was corrected at 690 nm (Infinite M200, Tecan, Grödig, Austria). The assay was performed at least in triplicate.

### 3.5. Production and Characterisation of Nanoparticles

Nanoparticles were formulated using the crude liquid extract of fucoidan (FUCe) obtained at 70 °C, 80 °C, 90 °C and 100 °C from the edible brown seaweed *Laminaria ochroleuca*. Their preparation involved polyelectrolyte complexation between the positively charged amino groups of chitosan (Sigma-Aldrich, Taufkirchen, Germany) and the negatively charged sulphate groups of the extract.

Chitosan (CS, low molecular weight, deacetylation degree ≈ 75–85%) was dissolved in acetic acid (1%, v/v). Several mass ratios (4/1; 1/1; 1/4) of FUCe/CS and CS/FUCe were used to prepare nanoparticles following two different protocols that basically differed in the order of addition of the components. Production of FUCe/CS nanoparticles was performed by adding 0.8 mL of CS at concentration varying between 0.625 and 10 mg/mL to 2 mL of FUCe at 1 mg/mL. In turn, CS/FUCe nanoparticles involved pouring 0.8 mL of FUCe (similarly, concentration between 0.625 and 10 mg/mL) in to 2 mL of CS at 1 mg/mL. The resulting dispersion was gently stirred in a magnetic stirrer for 10 min at room temperature. Each nanoparticle formulation was prepared at least in triplicate.

The prepared nanoparticles were characterised by their size (according to intensity), polydispersity index (PdI) and ζ potential, using a Zetasizer Nano ZS (Malvern PanAlytical, Malvern, UK). To do so, 40 µL of nanoparticles were mixed with 1 mL of Milli-Q water and introduced in an electrophoretic cell.

### 3.6. Mechanical Features of Alginate Fractions

Hydrogels formulated with aqueous dispersions of recovered alginates in the presence of CaCl_2_ (1% w/w) were made at a commonly employed biopolymer content (2.0 g L^−1^) according to Azevedo et al. [46]. Small amplitude oscillatory-shear testing of above systems was performed on a controlled-stress rheometer (MCR302, Anton Paar, Ostfildern, Germany). A sand blasted plate–plate (25 mm) was used as measurement geometry. Above hydrogels were loaded on the plate with a 1 mm gap and the sample edges were covered with paraffin oil. Before mechanical measurements, hydrogels were allowed resting (5 min) to promote thermal stabilisation. Firstly, stress sweeps were made as reported elsewhere [5] to determine the linear viscoelastic region (below 50 Pa). The viscoelastic behaviour (elastic, G’ and viscous, G’’ moduli) of tested samples was monitored with frequency at 25 °C within the linear viscoelastic region (at 20 Pa). Measurements were made at least in triplicate.

### 3.7. Statistical Analysis

Data were statistically evaluated using one-factor analysis of variance (ANOVA) using the PASW Statistics v.22 software (IBM SPSS Statistics, New York, NY, USA). Then, a Scheffé test was made to differentiate means (95% confidence, *p* < 0.05). Data for MTT assay, LDH assay and NP formulation were evaluated by one-way analysis of variance (ANOVA) using Dunn’s multiple comparisons test, Graphpad Prism^®^ statistical software (Version 6.01); differences were considered to be significant at a level of *p* < 0.05.

## 4. Conclusions

Different fractions of the brown seaweed *Laminaria ochroleuca* were obtained using an ecofriendly extraction technology, applying different temperatures between 70 °C and 100 °C. Alginate fractions and alginate-free liquid fraction extracts were studied. The composition and antioxidant capacity of the extracts were evaluated and the ability of the crude fucoidan extracts to be used as a nanoparticle matrix upon complexation with CS was evidenced. The cell viability of the extracts was evaluated in intestinal cells (HCT-116), showing higher cell inhibition for extracts obtained at lower temperatures (70 °C and 80 °C), which could be explained by the fucoidan:sulphate ratio. Future work should test the antitumoral activity of the extracts in other cell lines to evaluate the maintenance of this effect. With respect to nanoparticle production, it was verified that the order of addition of polymeric solutions and the used mass ratios translated into different physicochemical properties, permitting a certain tailoring of nanoparticle features. The temperature of extraction was found not to affect nanoparticle characteristics, which suggests that, based on the determined cytotoxic effect, extracts obtained at 70 °C and 80 °C from *Laminaria ochroleuca* warrant further study. Thus, these nanoparticles could be used in biomedical applications after associating candidate drug molecules.

## Figures and Tables

**Table 1:** Influence of the severity of the hydrothermal extraction of *Laminaria ochroleuca* on the total extraction yield and alginate yield, and on the composition and antiradical properties of the extracts obtained after alginate precipitation. Data values represent mean ± standard deviation (*n* ≥ 3); columns with different superscript letters are significantly different at the *p* ≤ 0.05 level.

T_max_ (°C)	Severity	pH	^1^ Extraction Yield (g/100 g Lo)	Alginate Yield (%)	Solid Content (%)	Protein Content (%)	PGE Content (%)	TEAC Value (%)
70	0.04	6.02 ± 0.05 ^b^	49.46	7.2 ± 0.8 ^c^	1.84 ± 0.01 ^c^	0.12 ± 0.01 ^a^	0.41 ± 0.02 ^b^	0.74 ± 0.02 ^c^
80	0.08	5.91 ± 0.01 ^a^	53.83	10.2 ± 0.6 ^b^	1.85 ± 0.01 ^c^	0.10 ± 0.01 ^a^	0.41 ± 0.11 ^b^	0.97 ± 0.03 ^a^
90	0.25	6.26 ± 0.01 ^a^	50.78	17.3 ± 1.2 ^a^	2.43 ± 0.05 ^a^	0.09 ± 0.02 ^a^	0.39 ± 0.04 ^b^	0.70 ± 0.04 ^c^
100	0.56	6.32 ± 0.01 ^a^	51.49	15.9 ± 0.9 ^a^	1.96 ± 0.01 ^b^	0.12 ± 0.01 ^a^	0.56 ± 0.16 ^a^	0.88 ± 0.01 ^b^

^1^ The variation was under 2%. PGE: phloroglucinol equivalents; TEAC: Trolox equivalent antioxidant capacity.

**Table 2:** Influence of the extraction temperature on the sulphate and monosaccharide composition of the extracts obtained after alginate precipitation. Data values represent mean ± standard deviation (*n* ≥ 3); columns with different superscript letters are significantly different at the *p* ≤ 0.05 level.

T_max_ (°C)	Sulphate Content (%)	Glu (%)	Xyl+Gal+ Man (%)	Rha (%)	Fuc (%)	Mannitol (%)	Acetyl Groups (%)
70	3.71 ± 0.08 ^a^	16.69 ± 0.88 ^a^	15.31 ± 0.21 ^b^	2.29 ± 0.01 ^d^	6.00 ± 0.14 ^c^	-	2.52 ± 0.21 ^c^
80	1.95 ± 0.03 ^c^	7.15 ± 0.25 ^c^	9.39 ± 0.55 ^c^	3.17 ± 0.39 ^c^	3.91 ± 0.20 ^d^	-	2.60 ± 0.59 ^c^
90	1.26 ± 0.02 ^d^	5.71 ± 0.48 ^d^	10.43 ± 0.48 ^c^	4.49 ± 0.23 ^b^	8.54 ± 0.20 ^b^	4.02 ± 0.20	3.90 ± 0.24 ^b^
100	2.23 ± 0.07 ^b^	10.70 ± 0.98 ^b^	20.61 ± 0.77 ^a^	6.86 ± 0.20 ^a^	18.01 ± 0.02 ^a^	-	6.30 ± 0.29 ^a^

Fuc: fucose; Gal: galactose; Glu: glucose; Man: mannose; Rha: rhamnose; Xyl: xylose.

## Supplementary Materials

The following are available online at https://www.mdpi.com/1660-3397/18/7/336/s1, Figure S1. Size distribution by intensity of the nanoparticles FUCe/CS (4/1 ratio, w/w) using the extract of *Laminaria ochroleuca* obtained by autohydrolysis at 70 °C (**a**), 80 °C (**b**), 90 °C (**c**) and 100 °C (**d**). Figure S2: Size distribution by intensity of the nanoparticles CS/FUCe (1/4 ratio, w/w) using the extract of *Laminaria ochroleuca* obtained by autohydrolysis at 70 °C (**a**), 80 °C (**b**), 90 °C (**c**) and 100 °C (**d**). Table S1. Polydispersity index (PdI) of the FUCe/CS and CS/FUCe nanoparticles produced with FUCe obtained at different temperatures (from 70 to 100 °C) and different ratios (4/1, 1/1 and 1/4). Data represent mean ± standard deviation (SD) (n ≥ 3).
