# Supplementary material for: Hydrothermal Processing of Laminaria ochroleuca for the Production of Crude Extracts Used to Formulate Polymeric Nanoparticles
Source: Mar Drugs. 2020 Jun 27;18(7):336. doi: 10.3390/md18070336 (PMC7401254; doi:10.3390/md18070336)
Supplement: Supplementary file 1 [file marinedrugs-18-00336-s001.pdf]

a)

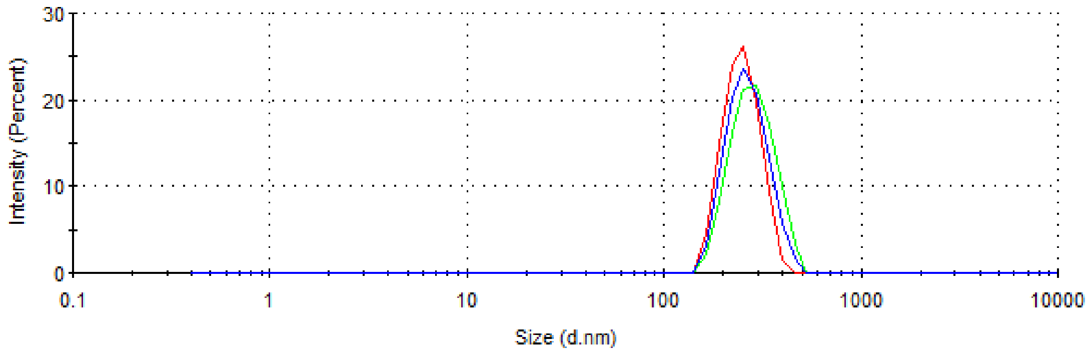

b)

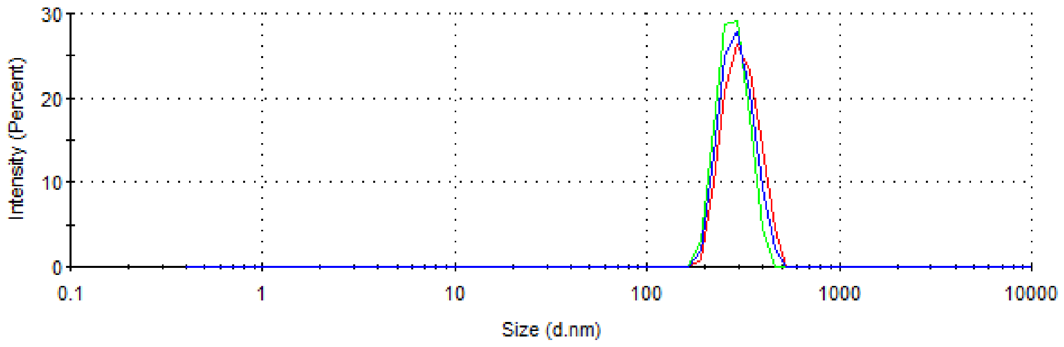

c)

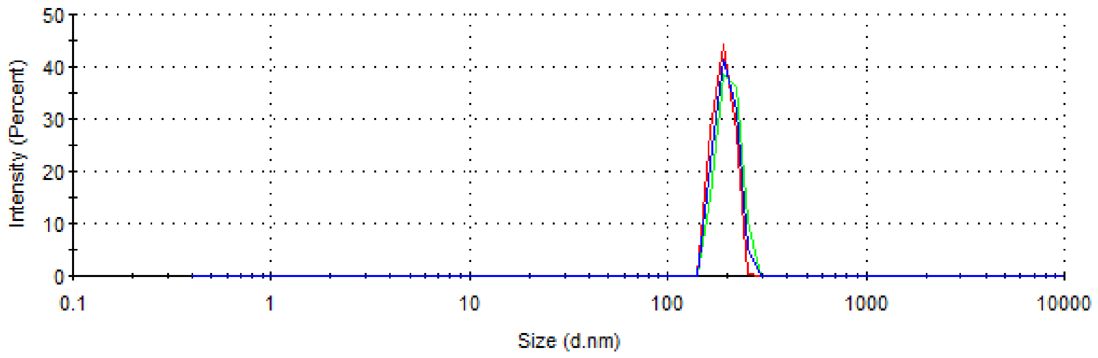

d)

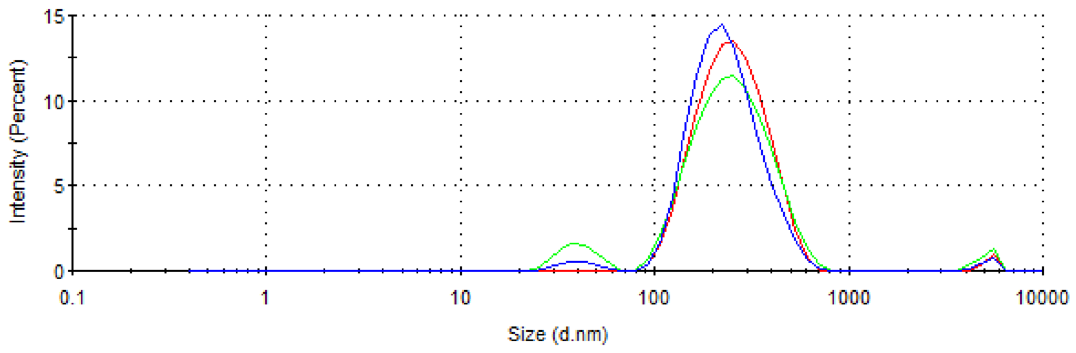

Figure S1. Size distribution by intensity of the nanoparticles FUCe/CS (4/1 ratio, w/w) using the extract of *Laminaria ochroleuca* obtained by autohydrolysis at 70 °C (a), 80 °C (b), 90 °C (c) and 100 °C (d).

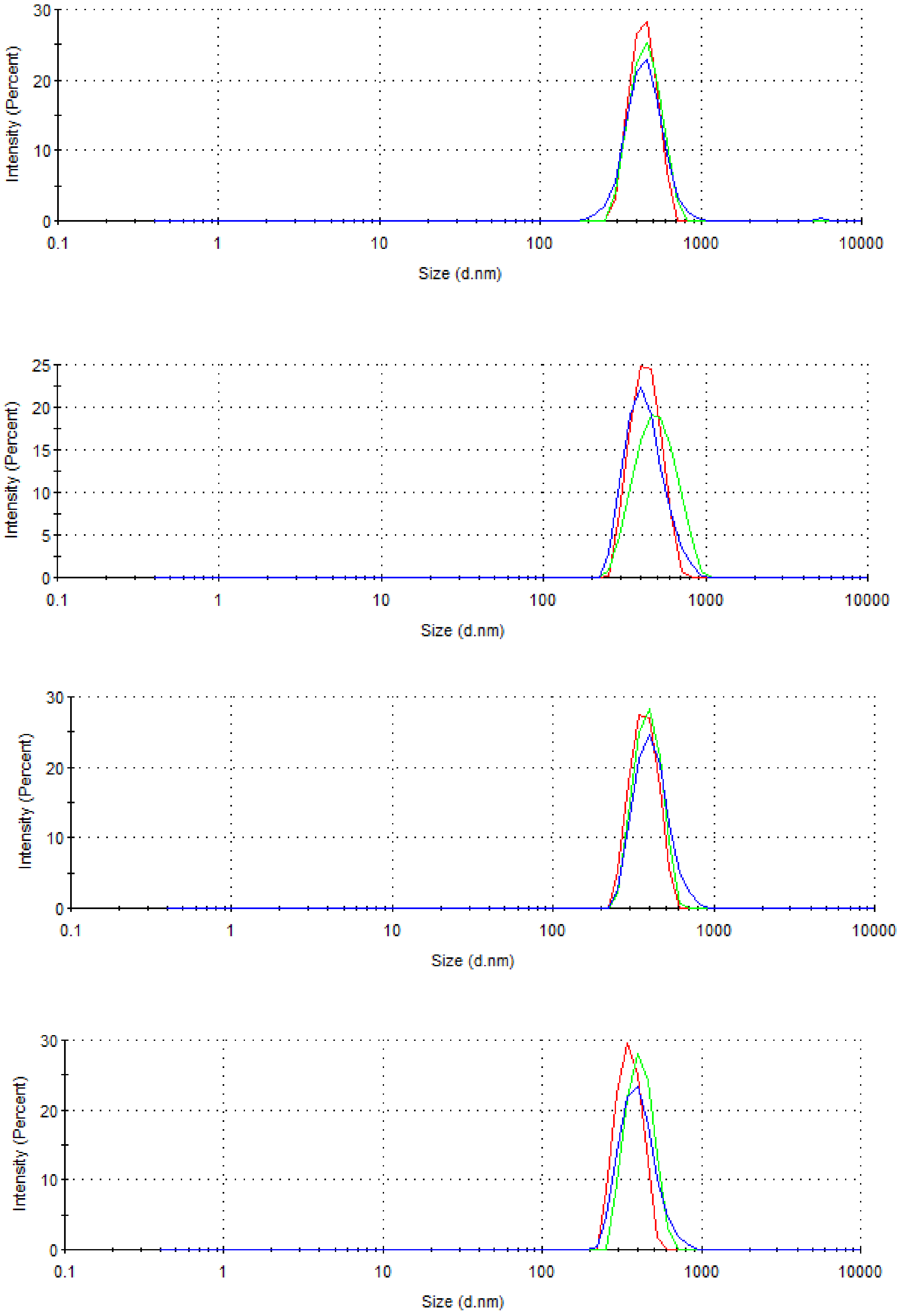

Figure S2. Size distribution by intensity of the nanoparticles CS/FUCe (1/4 ratio, w/w) using the extract of *Laminaria ochroleuca* obtained by autohydrolysis at 70 °C (a), 80 °C (b), 90 °C (c) and 100 °C (d).

Table S1. Polydispersity index (PdI) of the FUCe/CS and CS/FUCe nanoparticles produced with FUCe obtained at different temperatures (from 70 to 100 °C) and different ratios (4/1, 1/1 and 1/4). Data represent mean ± standard deviation (SD) (n ≥ 3).

		PdI	
**Temperature**	**Ratio**	**FUCe/CS**	**CS/FUCe**
70 °C	4/1	0.29 ± 0.06	0.44 ± 0.13
	1/1	0.44 ± 0.01	0.36 ± 0.11
	1/4	0.58 ± 0.02	0.28 ± 0.08
80 °C	4/1	0.35 ± 0.03	0.49 ± 0.07
	1/1	0.47 ± 0.01	0.42 ± 0.07
	1/4	0.49 ± 0.02	0.40 ± 0.01
90 °C	4/1	0.38 ± 0.12	0.57 ± 0.08
	1/1	0.57 ± 0.06	0.60 ± 0.10
	1/4	0.46 ± 0.06	0.34 ± 0.05
100 °C	4/1	0.40 ± 0.01	0.69 ± 0.10
	1/1	0.44 ± 0.06	0.50 ± 0.05
	1/4	0.49 ± 0.10	0.37 ± 0.07
